# Ecological speciation in a generalist consumer expands the trophic niche of a dominant predator

**DOI:** 10.1038/s41598-017-08263-9

**Published:** 2017-08-18

**Authors:** Stephen M. Thomas, Chris Harrod, Brian Hayden, Tommi Malinen, Kimmo K. Kahilainen

**Affiliations:** 1 Department of Environmental Sciences, University of Helsinki, PO Box 65, FI-00014 Helsinki, Finland; 2 Departments of Physiological Ecology & Evolutionary Genetics, Max Planck Institute for Limnology, D-24302 Plön, Germany; 3 Instituto de Ciencias Naturales Alexander Von Humboldt, Universidad de Antofagasta, Avenida Angamos 601, Antofagasta, Chile; 4 Biology Department, Canadian Rivers Institute, University of New Brunswick, Fredericton, NB E3B 5A3 Canada

## Abstract

Ecological speciation – whereby an ancestral founder species diversifies to fill vacant niches – is a phenomenon characteristic of newly formed ecosystems. Despite such ubiquity, ecosystem-level effects of such divergence remain poorly understood. Here, we compared the trophic niche of European whitefish (*Coregonus lavaretus*) and their predators in a series of contrasting subarctic lakes where this species had either diversified into four ecomorphologically distinct morphs or instead formed monomorphic populations. We found that the trophic niche of whitefish was almost three times larger in the polymorphic than in the monomorphic lakes, due to an increase in intraspecific specialisation. This trophic niche expansion was mirrored in brown trout (*Salmo trutta*), a major predator of whitefish. This represents amongst the first evidence for ecological speciation directly altering the trophic niche of a predator. We suggest such mechanisms may be a common and important – though presently overlooked – factor regulating trophic interactions in diverse ecosystems globally.

## Introduction

The integration of evolutionary biology and ecosystem science has recently been highlighted as one of the most underexplored areas in all of biology^1–3^. Although ecological factors have long been recognised as important drivers of speciation in natural populations, the extent to which species divergence may affect ecological processes has received less attention^3, 4^. However, interactions between within-species trait divergence and ecosystem function have been identified as a particularly fruitful avenue for research^3, 5^. This, coupled with recent evidence that ecological and evolutionary processes can occur over equivalent timescales, has drawn greater attention to this phenomenon^6, 7^. As a result, an emerging paradigm exploring such interactions has arisen over the past decade^1, 6^.

Contemporary ecosystem-level studies investigating reciprocal interplay between ecological and evolutionary process have largely focused on the top-down influence of diverging consumer species on their prey, although such studies have almost exclusively consisted of experimental manipulations, to variable outcomes^8–11^. There is, however, limited existing evidence for eco-evolutionary processes driving large-scale changes in complex natural ecosystems^5^. Although the capacity for such processes to have ecosystem-level consequences is generally acknowledged, the potential for such effects to be obscured by natural complexity has also been highlighted^5, 9, 11^. Identifying how these eco-evolutionary mechanisms manifest across trophic levels is an important step towards explaining potential ecosystem-level responses. However, this requires comparable ecosystems supporting contrasting diverging and non-diverging populations, which are rare in nature.

Although ecological speciation is a widespread phenomenon – occurring where disruptive selection pressures in contrasting environments lead to reproductive isolation of diverging sympatric populations – well-studied examples are typically confined to complex, diverse tropical ecosystems (e.g. Lake Tanganyika cichlids, *Anolis* lizards, *Geospizinae* finches)^4, 12, 13^. However, detection of the effects of evolutionary processes on the functioning of natural ecosystems almost certainly requires a focus on model organisms that are highly abundant, occur in relatively simple ecosystems, play a dominant role within their respective communities, show parallel divergence across multiple populations, and differ in functional traits which have effects at the level of the ecosystem^2^. Coregonid fishes may be ideal candidate organisms for such investigations, as adaptive radiation and ecological speciation are common across their distribution^14, 15^, various morphs show distinct patterns of resource use correlated with a series of readily quantifiable adaptive traits^16, 17^, and populations can reach very high densities within their supporting ecosystems^18^. European whitefish (*Coregonus lavaretus*), hereafter “whitefish”, largely satisfies the above criteria, showing the most pronounced patterns of resource polymorphism in its genus, with sympatric morphs displaying ecomorphological adaptations that facilitate exploitation of littoral, pelagic or profundal niches (refs 16, 19; see *Focal species* for more information). Moreover, as whitefish are often among the dominant prey species for piscivores across their range^20, 21^, they play a central role within their supporting food webs. Changes in functional traits such as relative rates of incorporation (and subsequent transfers) of matter and energy, driven by diverging patterns of resource use in this species, may therefore promote ecosystem-wide changes in nutrient cycling and other ecological processes^2^.

Evolutionary divergence of phenotypic traits associated with the trophic niche of abundant consumers may have particularly far reaching consequences for other trophic levels, and may be most pronounced where food webs are simple^1, 8, 10^. Further, ecological speciation processes may be amplified by interactions between the diverging species and their prey and predators, with the relative strength of such interactions mediating the magnitude of this response^1^. The central role of whitefish within their supporting food webs suggests that their divergence into multiple morphs has the potential to affect both their predators and prey, via both bottom-up and top-down mechanisms, respectively^17, 22^. The capacity for bottom-up effects of ecological speciation in a secondary consumer has received little attention to date, in favour of a focus on top-down processes^8–11^, but represents one of the major pathways by which evolutionary diversification may manifest within host ecosystems. This is likely to be particularly pronounced in relatively species-poor ecosystems, where predators have a limited selection of potential prey species. Within northern European lakes, brown trout (*Salmo trutta*) are widespread, have generally similar habitat requirements to their whitefish prey, and are often the dominant piscivore^20, 23^. As generalist predators, they are likely to show distinct, quantifiable responses to variability within their preferred prey, and thus may be prone to bottom-up influence in prey diversification.

In the present study, we explore the potential for ecological speciation in whitefish populations to drive ecosystem-level changes both in the niche of an abundant, widely-distributed top predator, and throughout the wider fish community, by combining short-term dietary and long-term stable isotopic analyses for quantification of trophic niche use. Using six subarctic lakes with either monomorphic (n = 3) or polymorphic (n = 3) whitefish populations (see *Focal species* for physical and ecological descriptions of morphs), we explore the specific hypotheses that: i) the phenotypic divergence of whitefish, allowing exploitation of three principal trophic niches (littoral, pelagic, profundal) in polymorphic systems, will drive differences in resource use, leading to a pronounced increase in trophic niche size relative to that of whitefish in monomorphic systems^16, 24^; (ii) whitefish, as the most abundant forage fish in the lakes, is a key prey species for piscivores^20, 21^, so any expansion of their trophic niche in polymorphic lakes should influence the niche of brown trout, a major whitefish predator in these systems; and (iii) effects on these two groups will elicit ecosystem-wide changes, increasing the complexity of the food web by increasing trophic linkages^2^, and promoting division of energetic pathways among littoral, pelagic and profundal sources.

## Results

**Figure 1:**
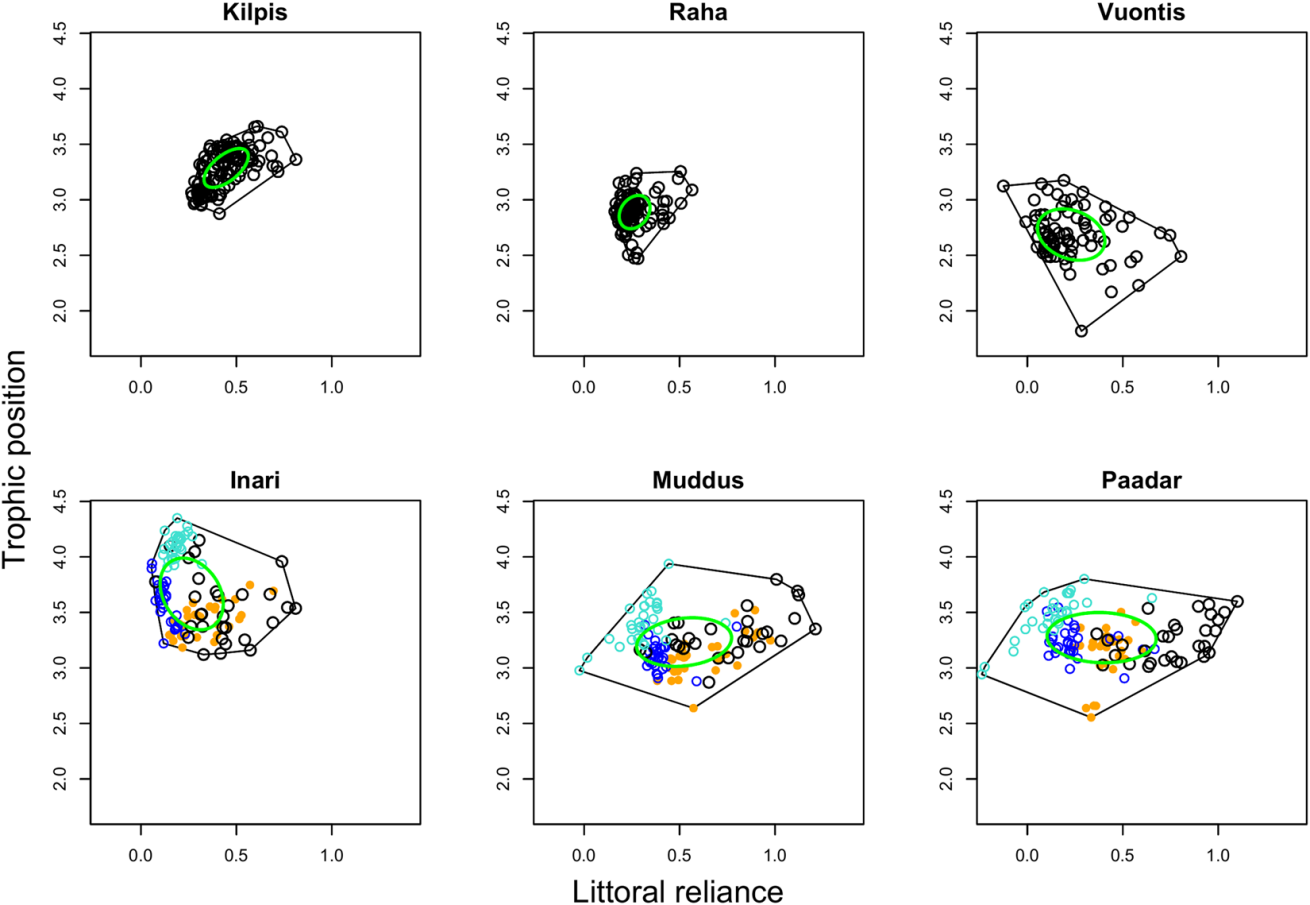
The total (convex hull) and core (SIBER ellipse) isotopic niche of monomorphic (top row) and polymorphic (bottom row) whitefish populations. Large sparsely-rakered (LSR) = black open circles; small sparsely-rakered (SSR) = open turquoise circles; large densely-rakered (LDR) = closed orange circles; densely-rakered (DR) = open blue circles.

**Figure 2:**
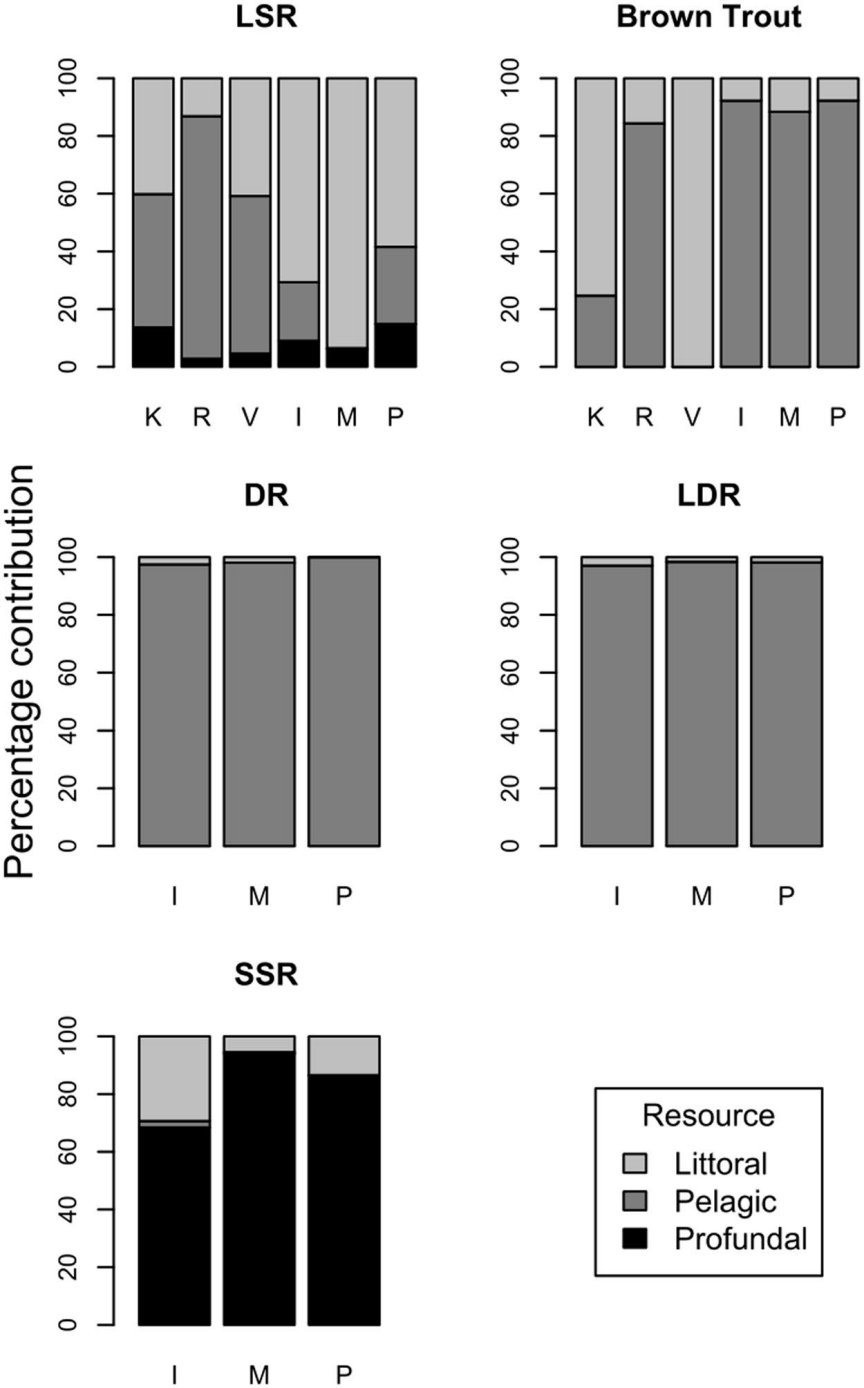
Mean proportion of littoral, pelagic and profundal diet items in stomach contents in focal species in lakes with monomorphic (K = Kilpis; R = Raha; V = Vuontis) and polymorphic (I = Inari; M = Muddus; P = Paadar) whitefish populations. See Fig. 1 legend for whitefish morph abbreviations.

The isotopic niche of whitefish was considerably expanded in polymorphic systems, compared to monomorphic ones (Fig. 1), and proportional contributions of habitat-specific prey to the diet of the LSR morph differed markedly between lake types (Fig. 2). In the polymorphic systems, the isotopic niche of whitefish populations was approximately 2.5 times larger than in the monomorphic systems, both in terms of total niche area (TA; mean: 0.88 vs. 0.36; t_4_ = −2.89; p = 0.04) and core niche area (SEA_c_; mean: 0.18 vs. 0.07; t_4_ = −3.54; p = 0.024). Individuals in polymorphic lakes were more widely dispersed in niche space compared to monomorphic lakes, as indicated by an increased mean distance to centroid in the former (0.31 vs. 0.19; t_4_ = −3.74; p = 0.02). Increasing niche size was related to trophic diversification among morphs, which showed pronounced differences in terms of both mean littoral reliance, and mean trophic position across all three polymorphic lakes (Fig. 1). This was driven by increased individual specialization to specific littoral, pelagic or profundal habitats and associated diet in polymorphic populations (Fig. 1; Fig. 2; Table S4), rather than a greater inclusion of novel prey taxa, and the mean number of dietary items used by whitefish in both lake types was identical (14.67 in both cases; t_4_ = 0; p = 1).

**Figure 3:**
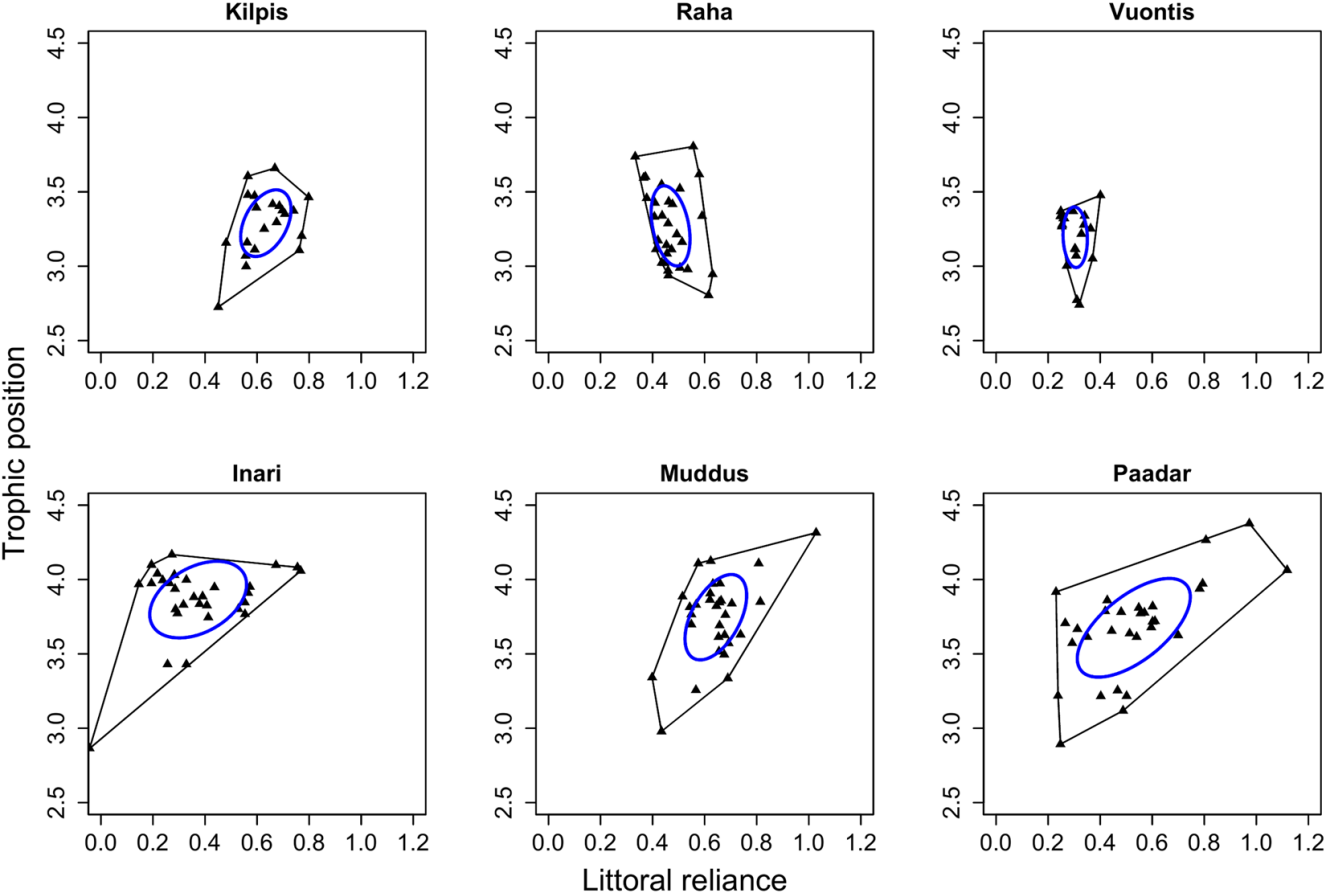
The total (convex hull) and core (SIBER ellipse) isotopic niche of brown trout in subarctic lakes with monomorphic (top row) and polymorphic (bottom row) whitefish populations.

Divergence in whitefish was associated with an enlargement in the isotopic niche of brown trout, which was approximately 2.5 – 3 times larger in lakes containing polymorphic whitefish compared to monomorphic whitefish (Fig. 3; mean TA = 0.47 vs. 0.15; t_4_ = −3.20; p = 0.033; mean SEA_c_: 0.14 vs. 0.05; t_4_ = −3.21; p = 0.033). Brown trout in polymorphic systems showed evidence for an increased tendency towards piscivory, being approximately half a trophic level higher in the food chain than in monomorphic systems (mean estimate: 3.76 vs. 3.25; t_4_ = −8.19; p = 0.001). Dietary analyses confirmed this pattern, documenting a shift from a mainly littoral diet in monomorphic lakes, to a predominantly pelagic diet with a greater proportion of fish in polymorphic lakes (Fig. 2, Table S4). This was the case in all monomorphic systems except one (Lake Raha), where an abundant introduced pelagic planktivore, vendace (*Coregonus albula*), was consumed heavily.

**Figure 4:**
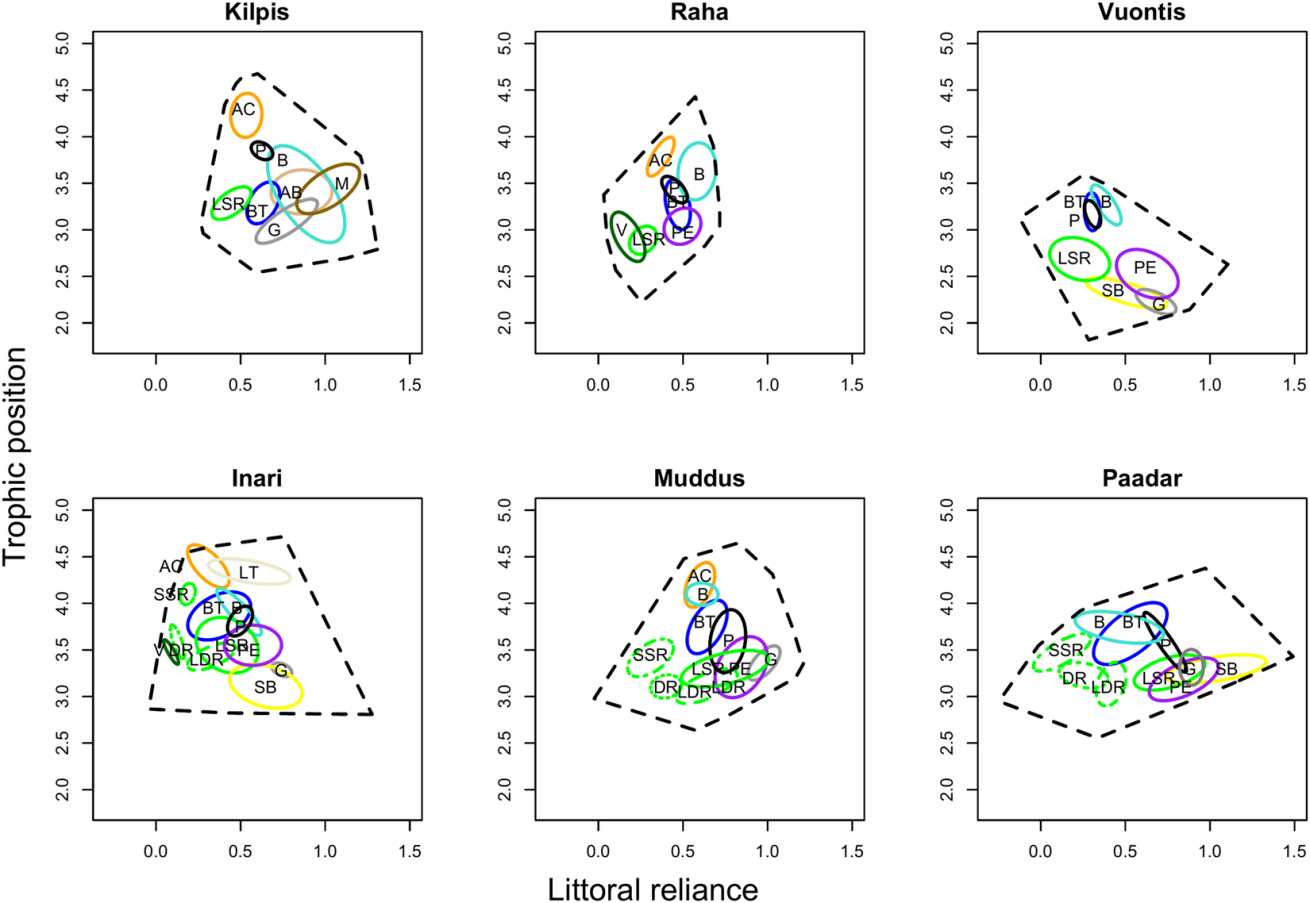
Isotopic niches of all fish species present within each lake. SIBER ellipses represent the core niche of each species, whilst convex hulls represent the total trophic diversity of the whole community. AB = alpine bullhead (*Cottus poecilopus*); AC = Arctic charr (*Salvelinus alpinus*); BT = brown trout (*Salmo trutta*); B = burbot (*Lota lota*); G = grayling (*Thymallus thymallus*); LT = lake trout (*Salvelinus namaycush*); P = pike (*Esox lucius*); PE = perch (*Perca fluviatilis*); SB = nine-spined stickleback (*Pungitius pungitius*); M = minnow (*Phoxinus phoxinus*); V = vendace (*Coregonus albula*). See Fig. 1 legend for whitefish morph abbreviations.

Despite ecological speciation in whitefish driving apparent changes in both their own niche and the niche of brown trout, differences at the level of the whole fish community were less pronounced (Fig. 4; Table 1). Though metrics such as mean niche size (mean TA: 1.66 vs. 1.22; t_4_ = −2.34; p = 0.079; mean SEA_c_: 0.30 vs. 0.23; t_4_ = −1.91; p = 0.129), mean trophic position (3.51 vs. 3.13; t_4_ = −1.78; p = 0.149), total number of trophic links (64.7 vs. 51.7; t_4_ = −1.71; p = 0.162) and food-web connectance (0.075 vs. 0.094; t_4_ = 2.57; p = 0.062) often varied considerably between polymorphic and monomorphic systems, none of these differences were significant at α = 0.05.

**Table 1:** Predator-prey food web metrics for communities in each study lake. All metrics are calculated from presence-absence species interaction data.

	Monomorphic			Polymorphic		
	Kilpis	Raha	Vuontis	Inari	Muddus	Paadar
Number of nodes	23	25	22	31	32	25
Number of links	56	57	42	71	70	53
Linkage density	2.44	2.28	1.91	2.29	2.19	2.12
Directed connectance	0.105	0.0912	0.087	0.073	0.068	0.085

## Discussion

Although ecological speciation has been documented in a variety of taxa^4, 12, 13, 25^ our data reveal for the first time how phenotypic divergence in a generalist consumer directly affects the flux of matter and energy through an ecosystem. We found that the trophic niche of polymorphic whitefish populations was nearly three times larger, on average, than monomorphic populations, supporting hypothesis (i). This enlargement of the isotopic niche was driven by increased dietary specialization among morphs towards littoral and profundal habitats. The expansion of isotopic niche size in polymorphic whitefish populations was mirrored in brown trout, with a directly comparable enlargement in trophic niche space, supporting hypothesis (ii). Contrary to our initial prediction, neither food-web topology, nor total isotopic niche size of the whole fish community, differed between monomorphic and polymorphic systems.

Our results demonstrate how increased specialization in polymorphic populations has led to more divergent patterns of resource use, occasionally spanning the full extent of observed pelagic-littoral niche space, as well as including profundal-derived energy sources. We reveal how increasing use of profundal and littoral habitats (by SSR and LSR whitefish, respectively) almost tripled the total isotopic niche of whitefish in polymorphic systems. This expansion appeared to be promoted by the emergence of more specialized pelagic morphs (i.e. DR and LDR), which acted to relegate ancestral LSR populations from their preferred pelagic habitats^16^. This process, where adaptive evolution promotes increased niche specialization compared to generalist ancestral populations, may be further strengthened and maintained by interactions between predators and prey^1, 11^. For example, increased specialization in diverging populations can act to modify the adaptive landscape, driving changes in prey size, density and population structure^1, 17^. Such interactions between consumers and resources have the potential to lead to feedbacks, further increasing adaptive evolution of both groups. In unison, these processes may lead to evolution of morphs displaying markedly different traits and ultimately reproductively isolated new species. Although very recent research has revealed how the presence of land-locked forms of North American alewife (*Alosa pseudoharengus*) can result in consequent phenotypic divergence in their predator species^26^, we believe that our results demonstrate for the first time how evolutionary processes can increase within-species trophic diversity and subsequently alter energetic pathways supporting a top predator.

Changes in the trophic niche of whitefish led to direct consequences for niche use by brown trout. The major mechanism of this shift appeared to reflect increased predation of whitefish in polymorphic systems, evidently due to greater prey availability as a result of the emergence of the DR morph, which, on average, has a considerably smaller body size and preferentially utilizes exposed pelagic habitats to a greater extent than the ancestral LSR morph^16, 21^. As brown trout are gape–limited, the presence of a size-appropriate pelagic forage fish likely facilitated their transition from a largely littoral and partly insectivorous diet towards pelagic piscivory^21^. This is further supported by heavy predation of introduced pelagic vendace in both polymorphic and monomorphic systems where they occur (i.e. Inari and Raha), as this species represents an almost exact trophic and morphological analogue of DR whitefish^21^. Similar shifts towards pelagic foraging typically arise in large lakes where the presence of an abundant, small-bodied pelagic species (e.g. smelt, *Osmerus eperlanus*, Arctic charr or sticklebacks) creates an additional resource for piscivores^27, 28^. We suggest that such patterns may be a common feature where ecological speciation occurs in lake fish, particularly where species diverge along littoral-pelagic gradients, as is common in for many northern hemisphere taxa^4, 29^.

Selective predation of morphs by piscivores, as observed here, may amplify natural selection, promoting further divergence within both prey species and their predators. For example, increased pelagic habitat use associated with zooplanktivory makes DR whitefish more susceptible to capture by brown trout, due to increased spatio-temporal overlap, increasing mortality rates, and thus promoting an accelerated life-cycle^22, 30^. However, the converse is true for SSR whitefish, which are able to reduce mortality rates by adapting to cold, dark, unproductive, profundal environments with limited predation exposure, promoting a long and slow life-history^21, 31, 32^. These patterns are evident in the contrasting age of maturity, growth rates and lifespan of both morphs^30, 31^. Although trophic cascade theory and biomanipulation experiments in aquatic ecosystems have recognized and revealed the importance of inherent interactions among trophic levels^28, 33^, there is increasing evidence of ubiquitous eco-evolutionary feedback loops which shape these interactions^1–3, 11^. The presence of such feedback loops in food webs are likely of key importance in shaping diversity, body size, abundance and life-history traits of both predator and prey, and may ultimately strengthen and maintain such ecomorphological specialization to distinct habitats. In lake fish, comparable mechanisms may be important in driving pelagic populations towards small size, early maturation and high density in across geographically diverse systems, with putative impacts across trophic levels.

Despite large-scale changes in the niche of whitefish and brown trout, we did not document any resultant consequences at the level of the whole fish community. Here, the potential lack of prey diversity in subarctic lakes, coupled with the generalist nature of the other fish species present, may result in the remaining community expanding their trophic niche to utilise most of the available isotopic niche space in monomorphic systems^34^. In the present study, our results suggest that whitefish trophic divergence was not due to increased inclusion of novel resources *per se*, but rather of increased species packing, whereby specialized morphs utilize only a subset of prey resources used by monomorphic populations. In the absence of polymorphic whitefish populations in monomorphic lakes, there may, therefore, be increased trophic specialisation among the remaining fish taxa, due to increased ecological opportunity facilitated by reduced competition for specific prey taxa. Alternatively, the simple food web topology metrics (which consider only binary presence-absence interactions, but not interaction frequency or taxa abundances that likely determine energy flow pathways in real ecosystems) used here may have resulted in us potentially overlooking more fine-scale community-level consequences of whitefish isotopic niche expansion, which might have been apparent if relative abundances were taken into account. Despite this, it may have been expected that any major consequences of interaction would have been captured in patterns of consumer isotopic composition, as this measure represents a time-integrated measure of trophic interactions^35, 36^. As a result, our findings may suggest that effects of species at the whole-community-level may be subtle, or may only manifest in extremely simplistic food webs. Such phenomena strongly warrant further investigation, but this may require the use of extremely depauperate study systems, where such effects are most likely to be detected.

Although the patterns we observed were both consistent and pronounced, the investigation was subject to some inherent caveats. Firstly, the study was not experimental, but this was a necessity as we aimed to explore functional changes *in situ* in the complex natural ecosystems where eco-evolutionary processes actually operate^1, 37^. A second potential issue was the low replication at the ecosystem-level (n = 3), as lakes containing all four whitefish morphs are scarce across subarctic Fennoscandia^24, 32^. However, we found a marked and consistent isotopic niche split along littoral-pelagic axis, a pattern that is very common in diverging fish populations, suggesting the mechanism may be important globally^4, 29, 37^. Lastly, there was a lack of true replicate lake ecosystems, as all lakes had slightly differing abiotic conditions and fish communities, which may have masked any effects mediated by whitefish divergence. However, the observed patterns of parallel divergence both between morphs and within their predators were strong across all lakes in spite of these differences, and the complementary evidence from stable isotopes and diet composition was mutually supportive, further strengthening the case for the likely generality of our findings.

### Conclusions

We demonstrate how ecological speciation in an abundant generalist consumer can dramatically extend patterns of resource use, resulting in the expansion of the trophic niche of a dominant predator. Moreover, we found that diversification of resource use and increased individual specialization in diverging populations gave rise to a novel bottom-up mechanism by which effects of ecological speciation in a consumer may manifest at the level of the ecosystem, in contrast to previously documented top-down processes^8–11^. This effect is likely to be particularly evident when a diverging species is highly abundant and acts as a central node within the food web, a pattern that has been documented in several groups of insects, amphibians and birds, in addition to fishes^4, 25, 29^. Given that ecological speciation occurs within many groups of organisms globally, this mechanism may be an important process regulating transfer of matter and energy within ecosystems.

## Methods

### Study lakes

We studied a total of six subarctic lakes with contrasting monomorphic (Lakes Kilpis, Raha, Vuontis) and polymorphic (Lakes Inari, Muddus, Paadar) whitefish populations, all located in northern Finland (SI Table 1). In brief, the area is characterised by clear-water lakes inhabited by a relatively species-poor (8–13 total fish species; see Table S1) and salmonid-dominated fish assemblages^24^. Large lakes in the region are deep and oligotrophic, well oxygenated year-round, and have an ice-free season lasting from May to November. The polymorphic whitefish lakes were selected to represent the widest level of divergence in subarctic Fennoscandia, whereas the monomorphic lakes were selected to present comparable surface areas and habitat availability. All lakes were broadly similar across a suite of abiotic variables and had a generally comparable fish fauna (SI Table 1), and sufficient size and bathymetric complexity to ensure pelagic/profundal/littoral habitat availability. Based on habitat availability, all lakes could theoretically support polymorphic whitefish populations^24^. However, a previous study^24^ focusing on a large suite of lakes with contrasting whitefish populations found that monomorphic lakes tend to have lower turbidity than polymorphic lakes, suggesting the latter are typically more productive than the former. This was true also of the monomorphic lakes sampled as part of the present study, which, on average, had lower turbidity, less humic colour and lower chlorophyll-a concentrations than the polymorphic lakes (SI Table 1).

### Focal species

Whitefish within the study region often exist as polymorphic populations, with up to four genetically and ecomorphologically distinct morphs co-existing in the same lake (Refs 16, 32, 38, 39 and SI Table 2). Morphs are identified based on head and gill raker (GR) morphology, and differ in body size, with each morph having distinct patterns of resource use^16, 19^. Within polymorphic lakes, densely-rakered (DR) whitefish feed on zooplankton in the pelagic zone; large densely-rakered (LDR) whitefish feed on zooplankton and terrestrial insects, mostly in pelagic habitats; large sparsely-rakered (LSR) whitefish utilize benthic macroinvertebrates in littoral areas; and small sparsely-rakered (SSR) whitefish feed on benthic macroinvertebrates in the profundal zone^16, 19, 32^. However, most lakes in the region host monomorphic populations of the LSR morph, which are generalist in resource use, and considered ancestral to the other morphs^16, 24, 38, 39^.

Fish communities in the study lakes are relatively species-poor and are dominated by coregonids. All major fish species were collected across the study lakes, to allow the isotopic niche of the entire fish community to be quantified, though some rare or very small-bodied species were not actively sampled (SI Table 3). All major species are native, except for the relatively recent introduction of vendace to Lake Inari and Lake Raha. Following introduction to both lakes, vendace underwent an initial population boom between 1985 and 1995, after which the species was established and populations stabilised and persisted^40^. Within their natural distributions, vendace and DR whitefish do not co-exist in the same lakes, a pattern also noted in their North American relatives, cisco (*Coregonus artedi*) and dwarf lake whitefish (*Coregonus clupeaformis*), likely due to pressures associated with competitive exclusion^41^. Due to the limited timescale since vendace introduction to these systems, we expected that ecologically relevant patterns in the whitefish morphs would remain relatively comparable to lakes without vendace (Fig. S1). Moreover, our sampling design aimed to limit any resultant bias by including one lake supporting introduced vendace in both monomorphic and polymorphic lake categories.

The brown trout is among the most common piscivore species in the study region (along with northern pike (*Esox lucius*), burbot (*Lota lota*) and Arctic charr (*Salvelinus alpinus*)), and was the only abundant predator species present across all study lakes. Brown trout is a widely-distributed generalist species that is able to use a wide range of available habitats within lakes, rivers and coastal areas^42^. In the study lakes, this species has a diverse diet, and is a major predator of coregonid fish^21^. Brown trout was therefore selected as a representative predator species in which to explore consequences of whitefish divergence.

### Sample collection

Fish and invertebrates were sampled during August and September between 1999 and 2014, at variable intervals (SI Table 3). Previous diet and isotopic studies across multiple years have indicated a high degree of temporal stability in resource use of all fishes in the study lakes^16, 20, 21^. A variety of fishing methods were used in order to ensure sufficient sample sizes. The majority of fish were caught using a gill net series comprising of eight 1.8 m high and 30 m long nets with mesh sizes (knot-to-knot) of 12, 15, 20, 25, 30, 35, 45 and 60 mm. Gill nets were set overnight in pelagic (0–5 m from the surface), littoral (<5 m depth) and profundal (>10 m depth) habitats. Additional burbot, grayling and pike were captured using long-line and angling. Small-sized pelagic species (vendace, DR whitefish) that are undersampled with gill nets were sampled using a small pelagic trawl (4 m high, 8 m wide, cod end of 3 mm mesh size; ref. 18). Some very small littoral species (3-spined stickleback, 9-spined stickleback, minnow) could not be actively targeted with the sampling methods used.

Zooplankton and benthic macroinvertebrates were sampled concurrently with fish to establish baseline stable isotope values. Benthic macroinvertebrates were collected from both littoral (1 m depth) and profundal habitats (10–40 m depth) using an Ekman grab (sampling area: 272 cm^2^). Zooplankton were sampled with vertical (0–15 m) tows using a 50-μm mesh plankton net. Bulk zooplankton samples were frozen at -20 °C, and benthic invertebrates were sorted to family prior to freezing.

Following capture, all fish were immediately removed from nets and euthanized by cerebral concussion. All fish were transported on ice to a field laboratory, where total length was measured to the nearest mm and wet mass to the nearest 0.1 g. Finally, the stomach of each individual was dissected for content analyses. A subset of fish from each species (number of individuals and proportional stomach content data are provided in SI Tables 3 and 4) was selected to provide representative sample of the population. From these fish, a sample of muscle tissue for stable isotope analysis was excised posterior to the dorsal fin, and frozen at -20 °C.

All fish were euthanized according to the Finnish Animal Conservation Law (32§ 9.8.2013/584), conducted by the permission holder (Kimmo Kahilainen). Fishing rights in Finland belong to the landowner according to the Finnish Fishing Law (5§ 27.5.2011/600) and the authors (Kimmo Kahilainen) obtained permits for research fishing from the landowner at each sampling site (Forest and Park Service, permit numbers 214/5713, 839/5713, 1386/5713). No ethical permission is required for described scientific sampling of fish and invertebrates according to the Finnish Animal Conservation Law (7§ 28.6.2013/498).

### Stable isotope analysis

Fish muscle and bulk invertebrate samples were freeze-dried for 48 h, ground, weighed (0.5–1.0 mg) and encapsulated into tin cups. Isotope samples were processed using an elemental analyser coupled to a continuous flow mass spectrometer (recorded analytical error: ~0.1‰ for δ ^13^C and ~0.3‰ for δ ^15^N). As lipids are ^13^C depleted relative to other major tissue constituents, variable lipid concentrations between species and individuals can obscure patters of resource use and preclude direct comparisons between taxa. As such, δ^13^C data from all fish were corrected for lipid content mathematically^43^.

### Stomach contents

Following dissection, stomach contents were identified and classified using a point method, whereby stomach fullness was estimated visually on a scale of 0 (empty) to 10 (fully extended), and the relative contribution (by volume) of each prey taxon to total stomach fullness recorded^44^. Prey items were identified to the lowest feasible taxonomic level (SI Table 4), based on the extent of digestion. Proportions of littoral, pelagic and profundal prey for whitefish morphs and brown trout were calculated from this detailed data (SI Table 4). Presence-absence data simultaneously obtained from stomach content analysis were used in subsequent food-web topology analyses.

### Sample-size standardization

In an attempt to remove potential bias due to differential sampling efforts between lakes, all sample sizes were standardised. For diet data, a random subsample of 50 individuals of each species (or fewer, where total sample sizes <50; see SI Table 3) was selected. However, in order to make total whitefish dietary niche size directly comparable among ecosystems a total of 200 individuals were randomly selected from each lake: 200 LSR whitefish were used in monomorphic systems, whilst 50 of each of the four morphs were selected in polymorphic systems. As sample sizes for isotopic data were generally smaller than those for diet data, 30 individuals (or, in a few cases, fewer; SI Table 3) were used. This sample size is considered adequate to represent the isotopic niche of a population^45^. Whitefish were assessed at the whole-population level, with 120 individuals selected from each lake, with these either equally split among morphs in polymorphic systems, or comprising solely of the LSR morph in the monomorphic systems.

### Statistical Analyses

All statistical analyses were conducted in R version 3.1.3^46^. Due to widely varying isotopic baselines across lakes, isotope data were transformed from lipid corrected δ^13^C and raw δ^15^N values into two trophic niche measures (littoral reliance and trophic level) using linear mixing equations. This allowed for representation of individuals in standardised niche space, rather than ecosystem specific “δ-space” (see SI Fig. 1 for untransformed data), as advocated previously^47, 48^. We used a modified version of this approach, generating proportional resource reliance and trophic position estimates for each individual using linear mixing equations^49^. Here, we assumed trophic enrichment factors (TEFs) of 0.4‰ for δ^13^C, and 3.4‰ for δ^15^N, respectively^50^. The resultant variables allowed for placement of all sampled consumers within trophic niche space, which was standardised to relative littoral and pelagic baselines across lakes. As resultant data were still continuous and bivariate in nature, we were able to calculate standard isotopic community metrics more typically conducted on raw δ values^51, 52^. Although a small percentage of fish consumers fell outside of the isotopic range of the invertebrate baselines in some lakes, we chose not to constrain calculated estimates, as we aimed to quantify the total trophic diversity within each species. Here, we assumed that the apparent disconnect between the isotopic composition of fish and their prey might have emerged for a variety of reasons: such differences are known to occur, for example, as a result of seasonal or spatial variability in isotope ratios of invertebrate prey^35^, or temporal discontinuities in isotopic turnover rates between small bodied invertebrates and larger fish species^36^, both of which may influence isotopic composition, and are difficult to disentangle in field settings. However, we were able to collect all major invertebrate groups occurring in fish diets across all lakes, ruling out major sources of variability caused by non-sampled prey items.

Following data conversion, six metrics of isotopic trophic diversity^51^ were calculated (using the *SIBER* routine of the *SIAR* package; ref. 52), for both the whole community and selected individual species, along with mean littoral reliance and trophic position. In addition, sample-size-corrected standard ellipse areas (SEA_c_) were calculated similarly. SEA_c_ estimates the core isotopic niche of the population, and is less sensitive to outliers compared to traditional convex-hull approaches^45, 52^, though the two methods provide complementary information on differing aspects of trophic niche size (i.e. TA = total niche area; SEA_c_ = core niche area). All ellipse fits were conducted using *SIAR*, and were based on 10000 posterior draws. Finally, t-tests were used compare resultant niche metrics between mono- and polymorphic systems. As Levene’s tests confirmed homogeneity of variances among groups in all cases, Student’s two-sample tests were used to make all comparisons.

The composition of stomach content data was analysed using the R package *cheddar* ^53^. Stomach content data were converted to presence-absence format and used to construct predator-prey interaction matrices for fish and their invertebrate prey in each lake. Following this, *cheddar* was used to calculate several common metrics of food-web topology: the total number of nodes and links within the food web, linkage density (i.e. the mean number of links per species) and the degree of directed connectance (i.e. the proportion of all possible links which are realised within the food web) were assessed for each lake.

## Electronic supplementary material

Supplementary Information

Table S4
