# Supplementary material for: Ecological speciation in a generalist consumer expands the trophic niche of a dominant predator
Source: Sci Rep. 2017 Aug 18;7:8765. doi: 10.1038/s41598-017-08263-9 (PMC5562900; doi:10.1038/s41598-017-08263-9)
Supplement: Supplementary file 1 — Supplementary Information [file 41598_2017_8263_MOESM1_ESM.pdf]

Ecological speciation in a generalist consumer expands the trophic niche of a dominant predator

Stephen M. Thomas[1], Chris Harrod[2,3], Brian Hayden[4], Tommi Malinen[1] and Kimmo K. Kahilainen[1]

[1]Department of Environmental Sciences, University of Helsinki, PO Box 65, FI-00014, Helsinki, Finland

[2]Departments of Physiological Ecology & Evolutionary Genetics, Max Planck Institute for Limnology, D-24302, Plön, Germany

[3]Instituto de Ciencias Naturales Alexander Von Humboldt, Universidad de Antofagasta, Avenida Angamos 601, Antofagasta, Chile

[4]Biology Department, Canadian Rivers Institute, University of New Brunswick, Fredericton, NB E3B 5A3, Canada

Correspondence: Stephen M. Thomas, Department of Fish Ecology and Evolution, EAWAG Swiss Federal Institute of Aquatic Science and Technology, Center for Ecology, Evolution and Biogeochemistry, Seestrasse 79, CH-6047 Kastanienbaum, Switzerland. E-mail: stephen.thomas@eawag.ch

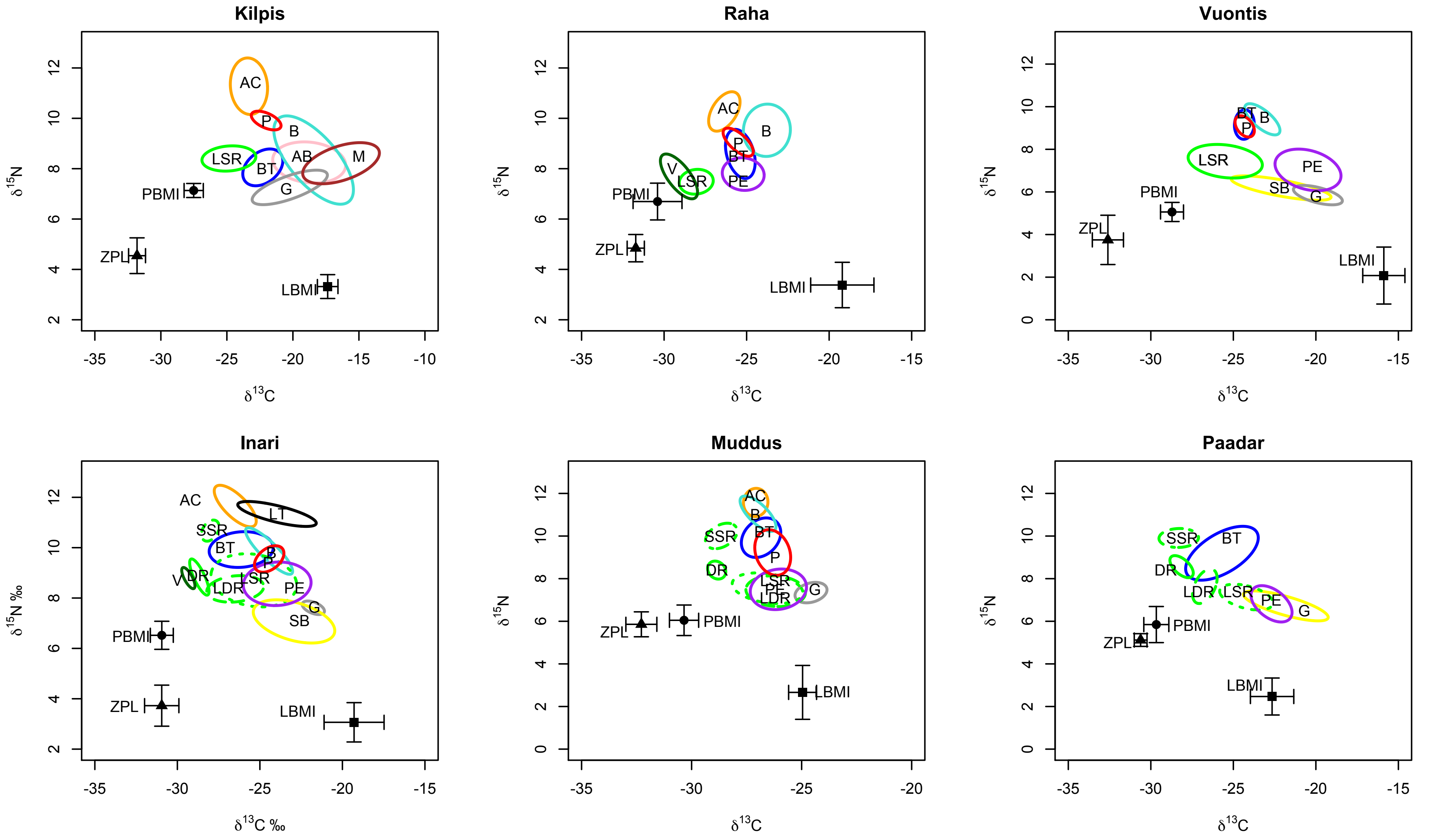
Kilpis
AC
P
B
LSR
BT
AB
M
G
PBMI
ZPL
LBMI
δ15N
δ13C
Raha
AC
B
P
BT
V
LSR
PE
PBMI
ZPL
LBMI
δ15N
δ13C
Vuontis
BT
B
P
LSR
PE
SB
G
PBMI
ZPL
LBMI
δ15N
δ13C
Inari
AC
LT
SSR
BT
B
P
V
DR
LDR
LSR
PE
G
SB
PBMI
ZPL
LBMI
δ15N ‰
δ13C ‰
Muddus
AC
B
SSR
BT
P
DR
LSR
PE
LDR
G
ZPL
PBMI
LBMI
δ15N
δ13C
Paadar
SSR
BT
DR
LDR
LSR
PE
G
PBMI
ZPL
LBMI
δ15N
δ13C

Fig. S1: Biplot of raw δ ^{13}C and δ ^{15}N for fish species and invertebrate baselines across all lakes. AB = alpine bullhead (*Cottus poecilopus*); AC = Arctic charr (*Salvelinus alpinus*); BT = brown trout (*Salmo trutta*); B = burbot (*Lota lota*); G = grayling (*Thymallus thymallus*); LT = lake trout (*Salvelinus namaycush*); P = pike (*Esox lucius*); PE = perch (*Perca fluviatilis*); SB = nine-spined stickleback (*Pungitius pungitius*); M = minnow (*Phoxinus phoxinus*); LSR = large sparsely-rakered whitefish; SSR = small sparsely-rakered whitefish; DR = densely-rakered whitefish; LDR = large densely-rakered whitefish; V = vendace (*Coregonus albula*); LBMI = littoral benthic macroinvertebrates; PBMI = profundal benthic macroinvertebrates; ZPL = zooplankton.

Table S1: Major biotic and abiotic data for all study lakes.

	Kilpis	Vuontis	Raha	Muddus	Paadar	Inari
Lake type	Monomorphic whitefish	Monomorphic whitefish	Monomorphic whitefish	Polymorphic whitefish	Polymorphic whitefish	Polymorphic whitefish
Location	69°00’N, 20°49’E	69°01’N, 27°04’E	68°45’N, 27°17’E	69°00’N, 26°50’E	68°52’N, 26°35’E	68°58’N, 27°40’E
Area (km^2)	37	11	23	48	21	1043 (32)†
Altitude (m a.s.l.)	473	151	132	146	144	118
Max depth (m)	57	31	46	73	56	92
Mean depth (m)	19.4	6.5	14.1	8.5	11.7	14.5
pH*	7.1	7.2	7.2	7.2	7.1	7.2
Tot P (μgl^{-1})	3	5	4	4	7	5
Tot N (μgl^{-1})	100	180	100	160	160	150
Chlorophyl-a	1.0	1.0	1.0	2.0	1.7	2.9
Secchi depth (m)	10	8	6.5	3	6	6
Colour (mg Ptl^{-1})	5	8	8	25	30	20
Compensation depth (m)	10	12	9	7	5	8
Percentage pelagic (%)	71	20	57	41	62	40 (37)
Number of fish species	8	9	11	10	9	13
Native fish species and morphs‡	LSR whitefish*<br>Arctic charr*<br>Brown trout*<br>Grayling*<br>Pike*<br>Burbot*<br>Minnow*<br>Alpine bullhead*	LSR whitefish*<br>Brown trout*<br>Grayling*<br>Pike*<br>Burbot*<br>Perch*<br>Minnow<br>9-sp. stickleback*<br>3-sp. stickleback	LSR whitefish*<br>Brown trout*<br>Arctic charr*<br>Grayling<br>Pike*<br>Burbot*<br>Perch*<br>Minnow<br>9-sp. stickleback<br>3-sp. stickleback	LSR whitefish*<br>SSR whitefish*<br>LDR whitefish*<br>DR whitefish*<br>Brown trout*<br>Arctic charr*<br>Grayling*<br>Pike*<br>Burbot*<br>Perch*<br>Minnow<br>9-sp. stickleback<br>3-sp. stickleback	LSR whitefish*<br>SSR whitefish*<br>LDR whitefish*<br>DR whitefish*<br>Brown trout*<br>Grayling*<br>Pike*<br>Burbot*<br>Perch*<br>Minnow<br>9-sp. stickleback<br>3-sp. stickleback	LSR whitefish*<br>SSR whitefish*<br>LDR whitefish*<br>DR whitefish*<br>Brown trout*<br>Arctic charr*<br>Grayling*<br>Pike*<br>Burbot*<br>Perch*<br>Minnow<br>9-sp. stickleback*<br>3-sp. stickleback
Stocked fish species	-	-	Vendace*	-	-	Vendace*<br>Lake trout*<br>Landlocked salmon

†Sampling within Lake Inari was confined to a single 32 km^2 bay (Nanguvuono) that is connected to the larger basin but relatively enclosed.

§Species' scientific names: Arctic charr = *Salvelinus alpinus*; Whitefish = *Coregonus lavaretus*; Brown trout = *Salmo trutta*; Grayling = *Thymallus thymallus;* Pike = *Esox lucius*; Burbot = *Lota lota*; Minnow = *Phoxinus phoxinus*; Alpine bullhead = *Cottus poecilopus*; Perch = *Perca fluviatilis*; Nine-spined stickleback = *Pungitius pungitius*; Three-spined stickleback = *Gasterosteus aculeatus*; Vendace = *Coregonus vandesius*; Lake trout = *Salvelinus namaycush*; Land-locked salmon = *Salmo salar*. Species marked with an "*" were captured and used in the study.

Table S2: Major morphological characteristics for the whitefish and brown trout populations within each study lake.

	Monomorphic			Polymorphic											
	Kilpis	Raha	Vuontis	Inari				Muddus				Paadar			
Morph	LSR	LSR	LSR	DR	LDR	LSR	SSR	DR	LDR	LSR	SSR	DR	LDR	LSR	SSR
Mean gill raker count	24.6	30.1	28.3	35.1	31.2	22.0	18.3	36.0	33.9	23.2	17.3	36.2	34.3	24.9	18.4
Max gill raker count	20	24	21	32	27	19	16	30	32	21	14	32	30	20	16
Min gill raker count	28	35	34	39	35	29	21	43	38	25	2	40	37	33	21
Mean length (cm)	25.05	18.65	18.70	12.16	28.68	26.89	21.93	13.97	30.30	25.20	17.70	14.93	29.49	21.02	18.83
Mean mass (g)	141.1	100.4	74.6	14.1	208.8	213.4	82.3	20.0	220.5	145.8	51.4	29.6	210.9	89.1	57.56
Littoral reliance	0.446	0.264	0.23	0.127	0.328	0.421	0.188	0.399	0.64	0.748	0.308	0.253	0.416	0.761	0.126
Trophic position	3.29	2.89	2.69	3.59	3.43	3.55	4.1	3.11	3.11	3.3	3.41	3.23	3.13	3.26	3.47
	BT	BT	BT	BT				BT				BT			
Mean length (cm)	27.29	38.23	39.98	37.15				40.50				39.00			
Mean mass (g)	215.8	699.6	734.9	683.1				743.6				764.1			
Littoral reliance	0.634	0.469	0.304	0.374				0.64				0.529			
Trophic position	3.29	3.27	3.20	3.86				3.75				3.68			

Table S3: Total sample size and the size of relevant subsamples used in each analysis type within each lake. Sampling years, including those where only invertebrate samples were taken. Years where samples were analysed for $\delta^{13}C$ and $\delta^{15}N$ are highlighted in bold.

	Monomorphic									Polymorphic								
Species	Kilpis (2002, **2005**, 2006, **2009**, 2010, **2011**, **2012**)			Raha (**2005**, 2006)			Vuontis (1999, 2000, **2004**, **2005**, **2007**)			Inari (**2009**, **2010**)			Muddus (1999, 2000, 2001, 2002, 2004, **2005**, **2006**, **2007**, **2011**, **2014**)			Paadar (**2004**, **2005**, **2012**, **2014**)		
	Tot.	Diet	SIA	Tot.	Diet	SIA	Tot.	Diet	SIA	Tot.	Diet	SIA	Tot.	Diet	SIA	Tot.	Diet	SIA
Alpine bullhead	19	15	17	–	–	–	–	–	–	–	–	–	–	–	–	–	–	–
Arctic charr	91	50	30	66	50	30	–	–	–	47	19	30	22	22	14	–	–	–
Brown trout	21	19	21	34	23	30	135	50	19	115	50	30	57	50	30	144	50	30
Burbot	53	50	30	13	11	13	21	8	21	31	–	30	20	15	20	21	19	9
Grayling	26	20	26	–	–	–	8	8	8	27	27	27	32	32	23	8	8	7
Lake trout	–	–	–	–	–	–	–	–	–	79	50	30	–	–	–	–	–	–
Minnow	12	9	12	–	–	–	–	–		–	–	–	–	–	–	–	–	–
Nine-spined stickleback	–	–	–	–	–	–	5	–	5	30	16	30	–	–	–	9	8	9
Perch	–	–	–	164	50	30	75	50	30	117	50	30	82	50	30	39	39	30
Pike	30	25	30	6	4	6	26	26	26	25	18	25	47	47	30	4	4	3
DR whitefish	–	–	–	–	–	–	–	–	–	104	50	30	87	50	30	85	50	30

LDR whitefish	–	–	–	–	–	–	–	–	–	47	34	30	59	50	30	61	50	30
LSR whitefish	520	200	120	272	200	105	189	189	80	94	50	30	73	50	30	92	50	30
SSR whitefish	–	–	–	–	–	–	–	–		131	50	30	70	50	30	63	50	30
Vendace	–	–	–	136	50	30	–	–	–	136	50	30	–	–	–	–	–	–
Total whitefish	520	200	120	271	200	105	189	189	80	376	184	120	289	200	120	301	200	120
Total fish	766	388	286	691	388	244	459	331	191	983	464	382	549	416	267	526	328	208
